# Biopesticide Trunk Injection Into Apple Trees: A Proof of Concept for the Systemic Movement of Mint and Cinnamon Essential Oils

**DOI:** 10.3389/fpls.2021.650132

**Published:** 2021-04-09

**Authors:** Pierre-Yves Werrie, Clément Burgeon, Guillaume Jean Le Goff, Thierry Hance, Marie-Laure Fauconnier

**Affiliations:** ^1^Laboratory of Chemistry of Natural Molecules, Gembloux Agro-Bio Tech, University of Liège, Gembloux, Belgium; ^2^Biodiversity Research Center, Earth and Life Institute, Université catholique de Louvain, Louvain-la-Neuve, Belgium

**Keywords:** essential oil, biopesticide, *Malus domestica*, trunk-injection, *Cinnamomum cassia*, *Menthas picata*, systemicity

## Abstract

The use of conventional pesticides is debated because of their multiple potential adverse effects on non-target organisms, human health, pest resistance development and environmental contaminations. In this setting, this study focused on developing alternatives, such as trunk-injected essential oil (EO)-based biopesticides. We analysed the ecophysiology of apple trees (*Malus domestica*) following the injection of *Cinnamomum cassia* and *Mentha spicata* nanoemulsions in the tree’s vascular system. Targeted and untargeted volatile organic compounds (VOCs) analyses were performed on leaf-contained and leaf-emitted VOCs and analysed through dynamic headspace–gas chromatography–mass spectrometry (DHS-GC-MS) and thermal desorption unit (TDU)-GC-MS. Our results showed that carvone, as a major constituent of the *M. spicata* EO, was contained in the leaves (mean concentrations ranging from 3.39 to 19.7 ng g_DW_^–1^) and emitted at a constant rate of approximately 0.2 ng g_DW_^–1^ h^–1^. *Trans*-cinnamaldehyde, *C. cassia*’s major component, accumulated in the leaves (mean concentrations of 83.46 and 350.54 ng g_DW_^–1^) without being emitted. Furthermore, our results highlighted the increase in various VOCs following EO injection, both in terms of leaf-contained VOCs, such as methyl salicylate, and in terms of leaf-emitted VOCs, such as caryophyllene. Principal component analysis (PCA) highlighted differences in terms of VOC profiles. In addition, an analysis of similarity (ANOSIM) and permutational multivariate analysis of variance (PERMANOVA) revealed that the VOC profiles were significantly impacted by the treatment. Maximum yields of photosystem II (Fv/Fm) were within the range of 0.80–0.85, indicating that the trees remained healthy throughout the experiment. Our targeted analysis demonstrated the systemic translocation of EOs through the plant’s vascular system. The untargeted analysis, on the other hand, highlighted the potential systemic acquired resistance (SAR) induction by these EOs. Lastly, *C. cassia* and *M. spicata* EOs did not appear phytotoxic to the treated trees, as demonstrated through chlorophyll fluorescence measurements. Hence, this work can be seen as a proof of concept for the use of trunk-injected EOs given the systemic translocation, increased production and release of biogenic VOCs (BVOCs) and absence of phytotoxicity. Further works should focus on the ecological impact of such treatments in orchards, as well as apple quality and production yields.

## Introduction

Apple *Malus domestica* Borkh is the most cultivated fruit crop worldwide, reaching a production of 84.7 million tonnes in 2016 and representing a gross product value of US $ 45.8 billion (FAOSTAT). As any other plant, apple trees are subject to abiotic and biotic stresses that cause important economic losses. Apple trees suffer from fungal, viral and bacterial diseases; insects; mites; and nematodes (Kellerhals et al., 2012). The rosy apple aphid, *Dysaphis plantaginea*, and the apple worm, *Cydia pomonella*, are amongst the most serious apple pests (Rousselin et al., 2017), whilst the main diseases are apple scab, powdery mildew, and fire blight caused by the fungi *Venturia inequalis* and *Podosphaera leucotricha* and by the bacteria *Erwinia amylovora*, respectively (Jamar et al., 2010). All these factors can impair production or marketable yields because apples do not fulfil the minimum quality criteria. Currently, the most applied delivery method for pest control is air-blast spray application of pesticides to the tree canopy (Damos et al., 2015). However, pesticide off-target drift can lead to adverse effects on non-target organisms. Over the last 50 years, biodiversity has been reduced by up to 50% in European bird species and by 20–30% in British and German flora (Geiger et al., 2010). Pesticides can cause environmental contamination and risks for human health through excessive residues on the fruit (Damalas and Eleftherohorinos, 2011). Additionally, pests can develop resistance to these pesticides, which usually contain a single active molecule (Alins et al., 2017). Altogether, this suggests that the plant protection product (PPP) mode of application selection is an economic and ecological challenge around the world. As a result of the negative perception of synthetic pesticides, causing negative effects on human health during and after application, and fears of their excessive residues in or on fruit, consumer demand for agricultural products without synthetic pesticide residues from excessive phytosanitary treatments has increased. This is why alternative solutions have been investigated, such as biological pesticides or biopesticides. An abundant body of literature is published each year concerning the prospect of plant essential oils (EOs) as active ingredients in the production of biopesticides (Campos et al., 2019).

The International Organisation for Standardisation (ISO) defines an EO as a “product obtained from vegetable raw material, either by distillation with water or steam, or from the epicarp of citrus fruits by a mechanical process, or by dry distillations.” Due to their biological activity, they have long been applied in cosmetics, therapeutics, and food applications (Hüsnü Can Başer and Buchbauer, 2015). The composition of EOs is highly variable and comprises a tremendous diversity of compounds. However, most of them belong to the terpenoids (mono- or sesqui-) or phenylpropanoids class of compounds, both of which have high lipophilicity and volatility, especially at room temperature. The secondary metabolites of EOs originate from methylerythritol phosphate and phenylalanine pathways (Rehman et al., 2016).

Some of the volatile organic compounds (VOCs) contained in EOs play a major role in plant defence mechanisms against bacteria, fungi, viruses, and herbivores (Bakkali et al., 2008). Therefore, much research has been performed to integrate these antibacterial, fungicidal, and insecticidal EOs as alternatives for sustainable agronomic practices, limiting environmental and health hazards. Indeed, due to their rapid degradation and since they are generally recognised as safe (GRAS), they represent an interesting alternative application of most synthetic conventional pesticides (Koul et al., 2008). Two EOs were used in this study:cinnamon EO (*Cinnamomum cassia* J. Presl) and mint EO (*Mentha spicata* L.). They both present well-documented biopesticidal activity (Singh and Pandey, 2018; De Clerck et al., 2020) due to their insecticidal and fungicidal (Muchembled et al., 2018; Lee et al., 2020) properties, which have already led to commercial product development (Isman et al., 2011; Isman, 2020). For example, mint EO has presented an inhibition concentration between 24 and 83 mg L^–1^on apple scab, depending on the strain (Muchembled et al., 2018). *C. cassia*, on the other hand, possesses a lethal dose 50 of 17.41 μl ml^–1^ on aphid *Myzus persicae* (Ikbal and Pavela, 2019).

Nevertheless, particular attention must be paid to the formulation of EO-based pesticides (Aćimović et al., 2020). A well-studied formulation could, on the one hand, counter the high volatility of EOs and ensure the prolonged release of the active substance and, on the other hand, attenuate potential phytotoxic effects (Moretti et al., 2002; Maes et al., 2019). EOs can impact many plant physiological processes (water status alteration, membrane integrity, respiration, and photosynthesis inhibition) through diverse modes of action, such as reactive oxygen species (ROS) induction and enzymatic or phytohormone regulation (Werrie et al., 2020). In this regards, chlorophyll fluorescence has been proven useful to evaluate plant vitality and response to abiotic stress (Kalaji et al., 2016). The application of EOs in apple tree may lead to phytotoxicity depending on the application method, concentration, and adaptive duration. For example, 7% of flowers were injured for clove oil in a thinning experiment for concentrations as low as 2% (Miller and Tworkoski, 2010). Fruit damages were also reported in postharvest treatment with savory, oregano, and thyme EOs at concentrations of 1–10% for the purpose of controlling *Botrytis cinerea* and *Penicillium expansum* (Lopez-Reyes et al., 2010). Nevertheless, fruit damage was not observed with thermal fogging treatment of lemongrass and citrus EOs at a concentration of 0.125% to control *B. cinerea* (Mbili et al., 2017). Therefore, the mode of EO application, the formulation and the selection of the active substance must be adapted for specific purposes and carefully evaluated.

Trunk injection is a method of applying chemicals directly to the vascular system of the tree after bark piercing, and the chemicals are then distributed systemically through the xylem tissue. This application method directly targets pests whilst reducing environmental exposure to pesticides and input quantities (Doccola and Wild, 2012; Wise et al., 2014). It has recently been experimented to fight fungi, such as apple scab, *Venturia inaequalis*, and powdery mildew, *Podosphaera leucotricha*, with disease severity reductions of 22–55 and 41.8–73.5% depending on the season and the product considered (potassium phosphites and synthetic fungicides) (Percival and Boyle, 2005; Aćimović et al., 2016b). A similar experiment on insect species [codling moth *Cydia pomonella* (L.), rosy apple aphid *Dysaphis plantaginea* (Passerini) and green apple aphid *Aphis pomi* (Passerini)] reported up to two seasons of control after a single injection of imidacloprid or emamectin benzoate (Wise et al., 2014). Spatial and temporal distributions of imidacloprid in leaves have been investigated (Aćimović et al., 2014), as well as residues to nectar and pollen, which were below the Environmental Protection Agency (EPA) threshold of 25 ng g^–1^ for imidacloprid (Coslor et al., 2019). Although the management of the injection timings may help to keep residue under the toxic limit, systemic resistance inducers have also been explored with the injection of acibenzolar-S-methyl (ASM) to induce systemic acquired resistance (SAR) and to control fire blight (Aćimović et al., 2015).

In the present study, we aimed to determine the distribution of trunk-injected EOs in young apple trees, thus proving their systemic movement by quantifying target VOCs, both within leaves and by aerial emissions. We also determined the impact of injected EOs on tree physiology by monitoring chlorophyll fluorescence and untargeted VOCs.

## Materials and Methods

### Essential Oils

The cinnamon EO (*C. cassia* J. Presl) and mint EO (*M. spicata* L.) used in this study were purchased from Pranarôm (Pranarôm & Herbalgem, Ghislenghien, Belgium). Before formulation of the EOs, the oil composition was analysed by gas chromatography associated with mass spectrometry (GC-MS). These analyses were carried out on a 7890A-5975C GC-MS equipped with an HP-5MS 30 m × 0.25 mm × 0.25 μm capillary silica column (Agilent Technologies Inc., Santa Clara, United States). The operating conditions were the following: helium flow of 1.0 ml min^–1^; the oven temperature was programmed at 40°C for 2 min, increased to 100°C at a rate of 5°C min^–1^, increased to 120°C at a rate of 3°C min^–1^, held for 3 min, increased to 220°C at a rate of 5°C min^–1^, and finally increased to 310°C at a rate of 15°C min^–1^. One microliter of a 1 mg ml^–1^ EO solution in hexane (HPLC grade, Merck KGaA, Darmstadt, Germany) was injected in splitless mode. The injector, quadrupole and MS temperatures were 250, 150, and 230°C, respectively. The mass spectrometer (MS) ran in electron impact (EI) mode at an electron energy of 70 eV. Mass spectra were acquired in the range of 30–400 atomic mass units (amu).

### Emulsion Formulations

To facilitate injection and diffusion of EOs in the tree vascular tissue, a water-soluble, stable, and homogenous EO emulsion was prepared. To prepare 100 ml of the 0.5% (v/v) EO/water emulsion, 2 ml of Tween 80 (CAS 9005-65-6, Merck KGaA, Darmstadt, Germany) and 20 ml of 100 mM ethylene diaminete traacetic acid (EDTA) (Titriplex III, Merck KGaA, Darmstadt, Germany) solution were added to 15 ml of water under constant agitation at 1,250 rpm. Water was then added to bring the final volume to 100 ml. After 5 min under constant agitation, the solution was then stabilised by high-speed homogenisation for 6 min at 9,500 rpm (Ultra-Turrax T25, IKA WERKE, Staufenim Breisgau, Germany) and by high-pressure homogenisation with eight cycles at 5,000 psi (FMC, Philadelphia, United States). The emulsion stability was checked by analysing the EO particle sizes and distribution in solution with a particle sizer (Beckman Coulter Delsa^TM^ Nano C Particle Analyser, California, United States).

### Biological Material

Experiments were performed on 2-years-old apple trees (*M. domestica* Borkh, cv “Jonagold” grafted on M26 rootstock) obtained locally (Serres de Sauvenières, Gembloux, Belgium). The trees were 155 ± 15 cm high and presented a trunk diameter of 2 ± 0.2 cm above the graft union. During the experimental phase, the plants were placed in an environmental chamber with controlled environmental conditions [21 ± 0.5°C, 62 ± 10% relative air humidity and 16:8 h light/dark periods and photosynthetically active radiation (PAR) of 50 μmol m^2^ s^–1^]. Plants were watered every day with 500 ml of water. They developed fully expanded leaves but were free of flowers or fruit.

### Trunk Injection System

**FIGURE 1:**
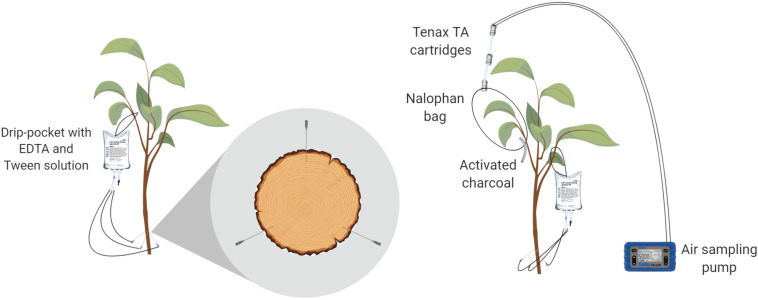
Laboratory trunk-injection device **(left)** and sampling of leaf-emitted volatile organic compounds (VOCs) **(right)**.

The trees used in the experiment were drilled right above the grafting union with holes that were 1 mm wide and 1 cm deep. Three trunk injection ports per tree trunk were created and were positioned at an equal distance from each other (each 120° of trunk radius). Each injection port was slanted upward at a 60° angle in relation to the trunk axis (Figure 1). Needles (BD vacutainer^®^ safety lock 23G, Becton Dickinson, New Jersey, United States) were inserted into the ports and connected on the other side to drip bags (Baxter^®^, Baxter International Inc., Deerfield, United States) filled with the solution injected (Figure 1). Four different treatments were tested using three biological replicates over a period of 96 h. The first two modalities were treated with EO emulsions (one with cinnamon oil and the other with mint oil), the third was a negative control (emulsion exempt of EOs) and the fourth was a blank treatment (no injection). To avoid cross-contamination, the treatments were delivered separately from each other at different times. Treatments were applied on different trees each time with a chamber ventilating period of 2 days.

### Volatile Organic Compound Sampling by Headspace Techniques

#### Leaf-Contained VOCs

Ten leaves were homogenously sampled at *t* = 0, 24, 48, 72, and 96 h on each replicate tree. Sampling was performed by cutting the leaves at their base and dipping them into liquid nitrogen before storage at -80°C prior to dynamic headspace–gas chromatography–mass spectrometry (DHS-GC-MS) analysis. A dry weight (DW) measurement was performed at the end of the experiment at 60°C until constant weight to obtain content results in ng g_DW_^–1^.

#### Leaf-Emitted VOCs

The headspace was sampled following the protocol for the volatile collection of aphid-infested leaves from an apple tree (Stewart-Jones and Poppy, 2006). Briefly, two Tenax TA^®^ 60/80 cartridges (Camsco^©^, Houston, United States) were attached to an inert polyethylene terephthalate (PET) bag (Nalophan^®^, Odometrics, Arlon, Belgium) enclosing a single branch. The trapping of emitted VOCs was performed by constant air sampling of 50 ml min^–1^ using a Gilian air sampling pump (Sensidyne^®^, St. Petersburg, United States) attached to the other side of the cartridges (Figure 1). Briefly, air enters the bag through the activated charcoal tube, loads in the VOCs and exits the bag through the Tenax TA cartridges, which capture VOCs. The bag and its connected cartridges were set up on each tree (*n* = 3) at *t* = 0 h. The cartridges were then replaced at *t* = 24, 48, 72, and 96 h and stored at −80°C prior to the GC-MS analysis. At the end of the experiment, all leaves enclosed in the bag were sampled and weighed. A DW measurement was also performed on these leaves at 60°C until constant weight to obtain results in ng g_DW_^–1^ h^–1^.

### VOC Analysis: Sample Preparation and GC-MS Analysis

Leaf-contained VOCs were analysed by DHS-GC-MS. Before dynamic headspace sampling (DHS), the leaves were ground (A11 basic grinder, IKA WERKE, Staufenim Breisgau, Germany) with liquid nitrogen. Then, 1 g of freeze-grinded leaves was put in a 20 ml screw cap vial (Gerstel^©^, Mülheiman der Ruhr, Germany), and 2 ml of a 20% (w/v) NaCl solution was added to create a salting out effect (Liberto et al., 2020). Afterward, the sealed vial was incubated in the dynamic headspace system at 35°C for 20 min (automated dynamic headspace DHS, Gerstel^©^, Mülheiman der Ruhr, Germany). The headspace was then dynamically transferred to a Tenax TA cartridge by applying 1,200 ml of nitrogen at a flow of 30 ml min^–1^. The cartridge was then drypurged at 50 ml min^–1^ for 4 min. The cartridge was then sent to the thermal desorption unit (Thermal Desorption Unit TDU 2, Gerstel^©^, Mülheiman der Ruhr, Germany) for GC-MS analysis. The thermal desorption parameters used were the same as those described below for leaf-emitted VOCs. Tenax TA^®^ porous polymers, based on 2,6-diphenyl-p-phenylene oxide, are widely used as an adsorbent in purge trap applications and plant headspace analysis due to their high versatility.

Leaf-emitted VOCs were analysed by TDU-GC-MS. Before thermal desorption, 1μl of 0.4 mg ml^–1^ 1-phenyloctane (CAS 2189-60-8, Merck KGaA, Darmstadt, Germany) in hexane was added to the cartridge by a multipurpose sampler (Multi-Purpose Sampler MPS, Gerstel^©^, Mülheiman der Ruhr, Germany). The addition of 1-phenyloctane as an internal standard (IS) allowed for semiquantification of the VOCs present on the cartridge. The VOCs were then thermally desorbed in the TDU and cryofocused in the cooled injection system (CIS) (Gerstel^©^, Mülheiman der Ruhr, Germany). The TDU temperature program was 40°C for 1 min and was increased to 280°C at a rate of 100°C min^–1^ and held for 5 min. CIS was mounted with a baffled glass liner and operated in solvent vent mode, and the temperature program was -60°C for 0.10 min, which was increased to 250°C at a rate of 12°C s^–1^ and held for 2 min following existing protocols (Delory et al., 2016; Durenne et al., 2018).

The analyses were carried out on a 7890A-5975C GC-MS equipped with an HP-5MS 30 m × 0.25 mm × 0.25 μm capillary silica column (Agilent Technologies Inc., Santa Clara, United States). The operating conditions were the following: helium flow of 1 ml min^–1^ and oven temperature 40°C for 2 min, which was increased to 220°C at a rate of 5°C min^–1^ and finally increased to 310°C at a rate of 15°C min^–1^ and held for 3 min. The quadrupole and MS temperatures were 150 and 230°C, respectively. The MS ran in EI mode at an electron energy of 70 eV. Mass spectra were acquired in the range of 30–400 amu.

For untargeted analysis, identification was based on comparison of the obtained spectra with the reference mass spectra from the NIST 17, Wiley 275 and pal 600 databases. Moreover, experimental retention indexes (RIs) were calculated using C_7_–C_30_ solutions and compared to literature RIs. Technical grade standards were injected to ensure identification (Nea et al., 2019; Tanoh et al., 2020). Semiquantification was performed using the following formula:

$$C\,o\,m\,p\,o\,u\,n\,d\,A\,c\,o\,n\,c\,e\,n\,t\,r\,a\,t\,i\,o\,n=\frac{c\,o\,m\,p\,o\,u\,n\,d\,A\,a\,r\,e\,a}{I\,S\,a\,r\,e\,a}*I\,S\,c\,o\,n\,c\,e\,n\,t\,r\,a\,t\,i\,o\,n$$

Detection and quantification of the major compounds of EO were performed in single-ion monitoring (SIM) mode. Based on the characterisation of the selected EOs, calibration curves in TDU-GC-MS using pure standards were established for each major component of the EO: (+)-carvone (CAS 2244-16-8, 99.9% purity, Supelco^©^, Missouri, United States) for mint and *trans*-cinnamaldehyde (CAS 14371-10-9, ≥99% purity, Merck KGaA, Darmstadt, Germany) for cinnamon oil. The 6-point calibration curves were established by injecting 1μl of the standard solution in hexane (Merck KGaA, Darmstadt, Germany). For (+)-carvone, ions 108 and 93 were selected as qualifiers, and ion 82 was selected as he quantifier. A calibration curve (*y* = 0.527*x*+0.020, *R*^2^ = 0.985) was established in triplicates between 1.50 and 861.05 μg ml^–1^. For *trans*-cinnamaldehyde, ions 132 and 103 were used as the qualifier, and ion 131 was used as the quantifier. A calibration curve (*y* = 0.628*x*+0.018, *R*^2^ = 0.989) was established in triplicates between 0.623 and 954.50 μg ml^–1^. The IS 1-phenyloctane was also used at a concentration of 400 μg ml^–1^.

### Chlorophyll Fluorescence Measurements

The potential phytotoxic effect of EOs on the photosynthetic efficiency of plants was evaluated by estimating the maximum quantum efficiency of photosystem II (Fv/Fm) with a fluorimeter (Handy PEA+, Hansatech Instruments Ltd., Norfolk, United Kingdom). For a healthy sample, this ratio is around 0.83 and lowers as plant stress increases, reaching 0.3 at the end of senescence (Bresson et al., 2018). Moreover, the maximum quantum yield of photosystem II has been used to evaluate foliar response after EO application (Synowiec et al., 2015, 2019). Measurements were performed at the same time of day for each time considered (*t* = 0, 24, 48, 72, and 96 h for each modality tested). Fv/Fm was assessed on three leaves randomly selected on each tree. Before the measurement, the leaves were dark-adapted for 20 min using leafclips. Fv/Fm measurements were then performed by exposing the leaves to light intensity of 3,000 μmol m^–2^ s^–1^.

### Statistical Analysis

The results from the targeted VOCs were visualised, and detailed nonparametric statistical analysis (Kruskal–Wallis test and Dunn’s test) was generated in Rstudio with ggstatplots (Patil, 2018). The untargeted VOC profiles, either contained or emitted, underwent several statistical analyses to understand the impact of the treatments performed on the apple trees. First, one-way analysis of variance (ANOVA) was performed for each VOC present in the profiles to understand which of them were significantly different between treatments using Tukey’s *post hoc* test. Descriptive statistics were coupled with principal component analyses (PCA) and heatmaps to visualise treatment effects and which VOCs they impact. All of these analyses were performed with metaboanalyst^1^ (Pang et al., 2020). Analysis of similarity (ANOSIM) and permutational multivariate analysis of variance (PERMANOVA) were performed between the different treatments. PERMANOVA tests the simultaneous response of one or more variables to one or more factors based on a similarity/distance matrix with permutation methods (Anderson, 2017). The ANOSIM and PERMANOVA were calculated in Rstudio (R 3.5.2 software, R Development Core Team, Boston United States) using the VEGAN package. ANOSIM and PERMANOVA were performed to establish if the contained and emitted VOC profiles were significantly impacted by the treatment. For fluorescence measurements, two-way repeated measures ANOVA was performed on the Fv/Fm dataset with treatments and time as a factor, followed by the pairwise *t-*test. A probability cutoff of α = 0.05 was applied for tests of significance in all statistical analyses and adjusted with the Bonferroni correction.

## Results

### Essential Oil Compositions and Formulations

GC-MS analysis of the EOs demonstrated that *C. cassia* oil was composed of 91.22% *trans*-cinnamaldehyde, and *M. spicata* was mainly composed of carvone (57.78%) and limonene (25.28%). A detailed composition can be found in the Supplementary Tables 1, 2. The EO compositions are similar to those reported before (Snoussi et al., 2015; Zhang et al., 2019). A stable nanoemulsion had a mean particle size diameter below 200 nm and a polydispersion index < 0.2.

### VOCs Spectra Analysis

#### Targeted Essential Oil Compounds

**FIGURE 2:**
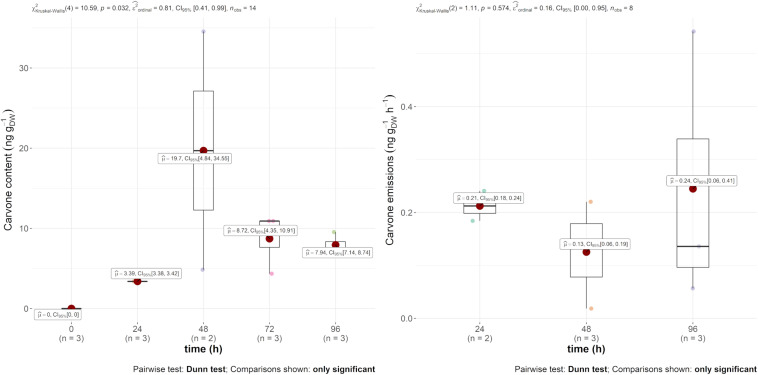
Boxplot of D-carvone contained (in ng g_DW_^–1^) in the leaves (left) and in the emissions (ng g_DW_^–1^ h^–1^) (right) over time after injection.

Regarding mint EO, the main compound, carvone, was found in both the emission and in the leaves, as displayed in Figure 2. The emission rate into the air was constant throughout the experiment at around 0.2 ng g_DW_^–1^ h^–1^. The leaf content, however, was more variable within and between 24 and 48 h. Indeed, the carvone content varied between 3.39 and 19.7 ng g_DW_^–1^, with a maximum 2 days after injection. However, as this compound was not found in the other treatments of the experiment, it demonstrates the systemic translocation of the trunk-injected mint EO.

**FIGURE 3:**
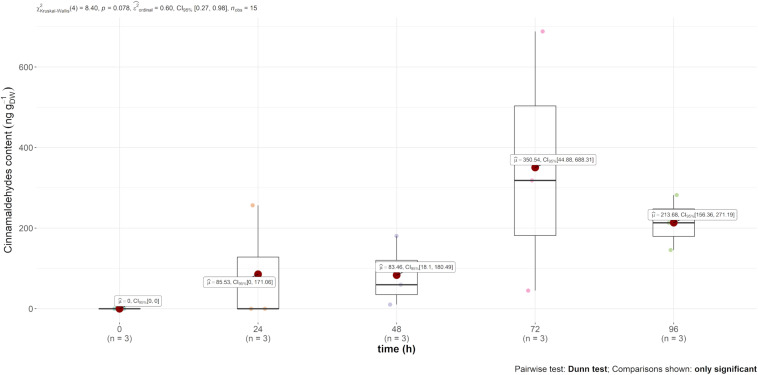
Boxplot of *trans*-cinnamaldehyde contained (in ng g_DW_^–1^) in the leaves over time after injection.

*Trans*-cinnamaldehyde, the main compound of cinnamon EO, was only recovered in the content of the leaf (and not in the air emission). However, this content was much higher in comparison to carvone, i.e., mint EO content, as observed in Figure 3, reaching 350 ng g_DW_^–1^ 72 h after injection.

#### Untargeted VOCs Emitted (TDU-GC-MS)

**FIGURE 4:**
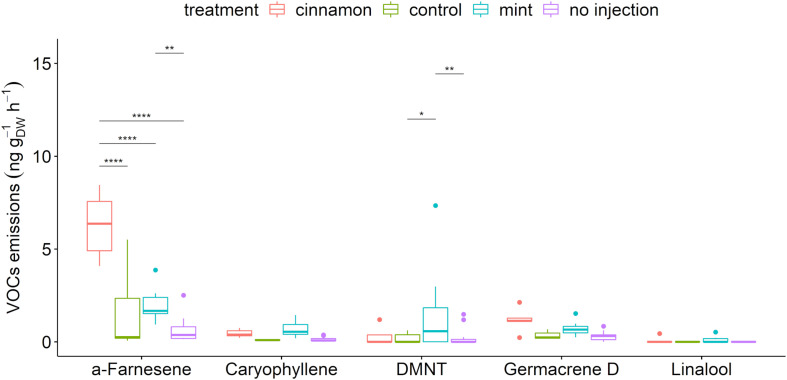
Boxplot of a selection of *Malus domestica* volatile organic compounds (VOCs) emitted (ng g_DW_^–1^ h^–1^) from plants injected with essential oils (EOs) and the control. The star symbols above the bars indicate a significant difference between the means (*P* < 0.05).

A total of 56 compounds were detected in the headspace emissions profiles of *M. domestica* trees belonging to the alkanes, alkenes, alcohols, aldehydes, aliphatic and aromatic esters, furanes, homoterpenes, ketones, monoterpenes, sesquiterpenes, and terpenoids (Supplementary Table S3). A selection of biogenic VOCs (BVOCs) that have a major biological role in the environment, such as pest attractant, attraction of pest-killing parasitic wasps, antennal response elicitor, or herbivory-induced plant volatile (Gershenzon and Dudareva, 2007; Hare, 2011; Souza et al., 2017), is presented in Figure 4. The apple trees that we injected with both EOs emitted the largest amounts of caryophyllene, linalool and germacrene D and significantly larger amounts of α-farnesene and (E)-4,8-dimethyl-nonatriene (DMNT).

**FIGURE 5:**
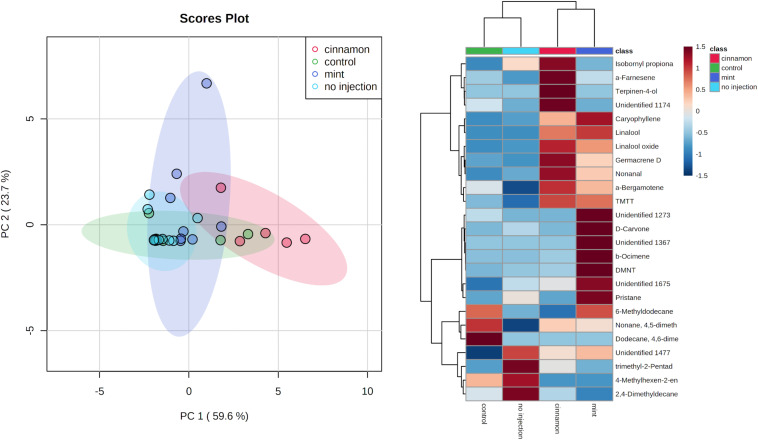
Principal component analysis (PCA) **(left)** and heatmap of the top 25 contributors merged by group **(right)** of *Malus domestica* volatile organic compound (VOC) emissions generated on metaboanalyst after data processing.

Multivariate analysis of the emitted VOC profiles performed by PCA captured 83.3% of variance in the first two dimensions (Figure 5). VOC profiles of EO-injected trees separated well from the control and no injection treatment. On the other hand, as can be observed in the heatmap (Figure 5), some compounds are produced for both oils, such as caryophyllene, germacrene D, bergamotene, (E,E)-4,8,12-trimethyltrideca-1,3,7,11-tetraene (TMTT) and linalool, whereas some of them are specific to a particular oil. Indeed, cinnamon-oil-injected trees emitted more terpinen-4-ol, α-farnesene, and trees injected with mint oil emitted more DMNT and β-ocimene. Amongst the compounds previously mentioned, linalool, germacrene D and terpinen-4-ol are found in mint EO, and caryophyllene is found in cinnamon EO, but as minor compounds at concentrations below 1%.

The VOC emission profiles were significantly impacted by the treatment. ANOSIM revealed significant structural differences for VOC profiles between treatments, with some overlapping (*R* = 0.281, *p* = 0.002). On the other hand, PERMANOVA performed on the same data set revealed similar outcomes for comparisons between treatment (*F* = 3.9517, *p* = 0.001^∗∗∗^). Pairwise PERMANOVA yielded significant differences for multiple comparisons in all cases, except for the no injection-control and mint-control, as shown in Table 1.

**TABLE 1:** Pairwise permutational multivariate analysis of variance (PERMANOVA) comparisons for volatile organic compounds (VOCs) emissions between treatment.

	**No injection**	**Cinnamon**	**Control**
Cinnamon	0.006**	–	–
Control	0.400	0.013*	–
Mint	0.006**	0.006**	0.253

The asterisks indicate significant differences: *P **≤**0.05, **P ≤ 0.01.

#### Untargeted VOCs Contained (DHS-GC-MS)

**FIGURE 6:**
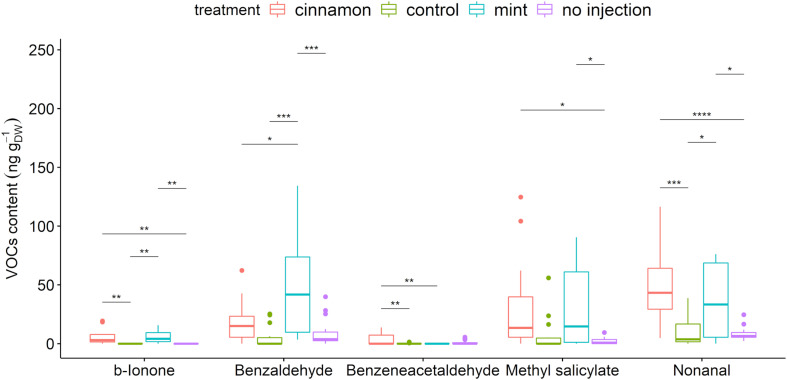
Boxplot of a selection of *Malus domestica* volatile organic compounds (VOCs)content (ng g_DW_^– 1^) from plants injected with mint and cinnamon essential oils (EOs) and the control. The asterisk symbols above the bars indicate a significant difference between the means (*P* < 0.05).

A total of 67 compounds were detected in the VOCs contained within leaves. These compounds belong to the alcohols, aldehydes, alkadienes, alkanes, aromatic and aliphatic esters, fatty acid esters, homoterpenes, and ketones (Supplementary Table 4). Injection of EOs significantly increased methyl salicylate, benzaldehyde, benzeneacetaldehyde, β-ionone, and nonanal (Figure 6). Amongst those compounds, only benzaldehyde was found in the cinnamon EO but also as a minor compound below 1%.

**FIGURE 7:**
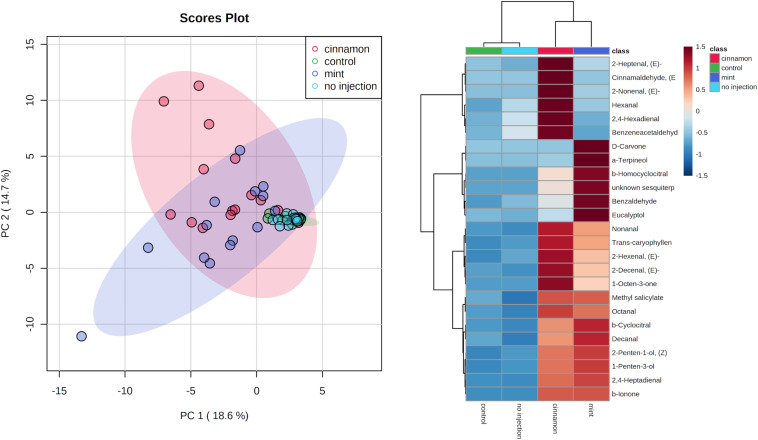
Principal component analysis (PCA) (left) and heatmap of top 25 contributors merged by group (right) of *Malus domestica* volatile organic compounds (VOCs) contained generated on metaboanalyst software after data centring processing.

VOC profiles for EO-treated trees were much more dispersed in comparison to the control and no injection treatments (Figure 7). As for the emitted VOCs, it seems from the heatmap that some compounds increased for both oil treatments, such as decanal, caryophyllene and 1-penten-3-ol. Some increases were specific, such as numerous aldehydes for cinnamon oil (2-heptenal, 2-nonenal, 2,4-hexadienal) and terpenes for mint oil (α-terpineol, eucalyptol, β-homocyclocitral). With regard to the EO composition, only α-terpineol was found in trace amounts within the mint EO at 0.25%.

ANOSIM revealed significant differences for VOC profiles between treatments, with some overlapping between group (*R* = 0.2712, *p* = 0.001). PERMANOVA analysis of the same dataset revealed similar outcomes for comparisons between treatments (*F* = 7.3673, *p* = 0.001^∗∗∗^). Finally, pairwise PERMANOVA revealed significant pairwise differences between all treatments, except for the control–no injection and cinnamon–mint, as shown in Table 2.

**TABLE 2:** Pairwise permutational multivariate analysis of variance (PERMANOVA) comparisons for volatile organic compounds (VOCs) contained between treatments.

	**No injection**	**Cinnamon**	**Control**
Cinnamon	0.006**	–	–
Control	0.585	0.006**	–
Mint	0.016*	0.151	0.022*

The asterisks indicate significant differences: *P ≤ 0.05, **P ≤ 0.01.

### Chlorophyll Fluorescence

**FIGURE 8:**
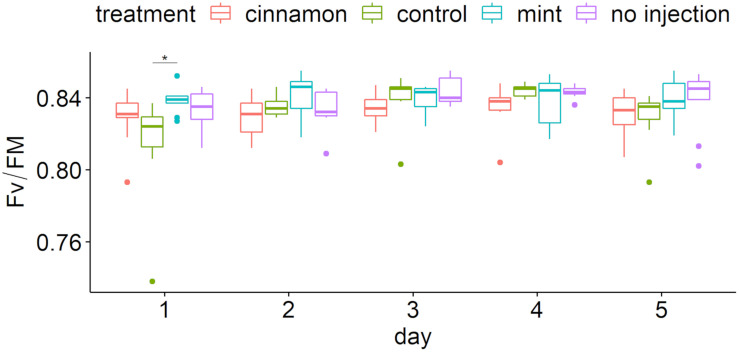
Maximum quantum yield of photosystem II (Fv/Fm) boxplot per treatment during time after injection. The star symbols above the bars indicate a significant difference between the means (*P* < 0.05).

Maximum yields of photosystem II (Fv/Fm) over time are presented in Figure 8. Chlorophyll fluorescence showed that most values were located between 0.80 and 0.85, implying that the trees maintained good ecophysiological performances throughout the experiment (Figure 8). Two-way repeated measure ANOVA revealed a significant impact of factors (treatment: *F* = 4.759, *p* = 0.003, ges = 0.082; day: *F* = 4.782, *p* = 0.001, ges = 0.107) without interaction (*F* = 1.59, *p* = 0.099, ges = 0.107). Pairwise comparison demonstrated significant differences only at day 1 between treatment (Figure 8) and only for the control between 24 and 48 h and 24 and 96 h.

## Discussion

Taken altogether, our results demonstrate, for the first time, the systemic translocation of trunk-injected EOs in apple plants. Carvone increased in the leaf content and was emitted at a constant rate, and *trans*-cinnamaldehyde content increased in the leaves but was not found in detectable amounts in the air emissions. The strong spatial heterogeneity combined with the relatively small sampling may also contribute to the variability of the results. However, it appears that the EO translocation within apple tree tissues and its diffusion in ambient air must be conditioned by its own physicochemical properties. Amongst those properties, vapour pressure, organic carbon–water partitioning coefficient (Ko/c), and the octanol water partition coefficient (Ko/w) may explain the differences observed between carvone and *trans*-cinnamaldehyde (Doccola and Wild, 2012; Montecchio, 2013; Aćimović, 2014). Out of these two, *trans*-cinnamaldehyde was the molecule with the smallest vapour pressure of 15.3 and 3.853 Pa at 25°C for carvone and *trans*-cinnamaldehyde, respectively (European Chemical Agency, 2020). This molecule, following Henry’s law, has a smaller tendency to volatilise and hence accumulates in the leaves. Moreover, from a histological point of view, they diffuse slowly through aqueous phases in the mesophyll, lipid bilayer membranes and internal airspace (in the substomatal cavity) before release through the stomata (Calfapietra et al., 2013). This diffusion is conditioned following the compounds’ octanol water partition coefficients (Ko/w), which are 2.7 for carvone and 2.1 for *trans*-cinnamaldehyde. It is worth mentioning that other phenomena could concurrently take place, such as the potential transformation or degradation of these xenobiotic compounds by the apple plants. Diverse mechanisms such as reduction/oxidation, esterification or conjugation with carbohydrates (glycosylation) or glutathione (glutathionylation) were demonstrated *in planta* for numerous GLVs and terpenes (Matsui et al., 2012; Rivas et al., 2013) and by diverse microorganisms (Asakawa et al., 2018). This was specifically demonstrated for *Arabidopsis* aldehydeoxidase 4 (AAO4) extracted from *Arabidopsis thaliana* developing seeds that could convert *trans*-cinnamaldehyde *in vitro* (Ibdah et al., 2009).

In addition to the established systemic circulation of carvone and *trans*-cinnamaldehyde, it is most interesting to look at the modification of other VOCs in the emission profiles that can strongly impact trophic interaction within ecosystems. BVOC emissions can mediate herbivore interactions (Trowbridge and Stoy, 2013). Within the framework of this discussion, one should bear in mind that numerous factors can influence apple tree VOC emissions, including meteorological (Vallat et al., 2005), circadian (Giacomuzzi et al., 2017), physiological (Zeng et al., 2017), and phonological (Casado et al., 2006), as well as interactions with herbivores (Suckling et al., 2012) or fungi (Souleyre et al., 2019). However, systemic release of induced volatiles also occurs in plants in the case of insect feeding to recruit natural enemies. The homoterpenes DMNT and TMTT, the monoterpenes ocimene and linalool and these squiterpenes farnesene and caryophyllene are a shared response to herbivores in diverse plant systems (Paré and Tumlinson, 1999; Holopainen and Gershenzon, 2010). Therefore, modification of emitted VOCs, such as those observed in our work, may alter trophic interactions with regard to chemical ecology. Moreover, germacrene-D, α-farnesene and methyl salicylate may have resulted from SAR activation by the injected EOs, since SAR has been detected after trunk injection of SAR activators (Aćimović et al., 2015). Indeed, monoterpenes have been acknowledged to support SAR amongst different plants (Riedlmeier et al., 2017). The elicitation of resistance in young apple trees by acibenzolar-S-methyl was observed to specifically increase the production of the compounds that were effective against rosy apple aphids and *Erwinia amylovora* (Aćimović et al., 2015; Warneys et al., 2018). Moreover, *Cinnamomum zeylanicum* oil and *trans*-cinnamaldehyde were proven to be efficient against Alternaria brown spot in tangerine by direct effects and resistance induction (Perina et al., 2019). A prospective molecular tool such as quantitative real-time PCR to detect changes in expression levels of genes involved in plant defence mechanisms may prove useful to challenge this hypothesis (Dugé De Bernonville et al., 2014; Aćimović et al., 2015). The plant defence responses include other mechanisms, such as cell wall fortification, antimicrobial compounds such as pathogenesis related (PR)protein productions, phytoalexins or reactive oxygen species (ROS) (Marolleau et al., 2017). Phytoalexins include diverse plant secondary metabolites biosynthesised in response to pathogens and certain abiotic stresses. In the subtribe Malinae of the Rosaceae family, the phytoalexins biphenyl and dibenzofuranare are produced upon pathogen attack (Chizzali and Beerhues, 2012) and after elicitor-treated cell cultures (Saini et al., 2019; Teotia et al., 2019). The production of phytoalexins following treatment with EOs could also be an interesting prospect in order to determine and clarify the defence induction potential of these compounds as well as their potential impact on pathogens.

The results presented in this work clearly exposed the possibility that EO application could trigger different physiological processes within plants, leading to other BVOC emissions. Some compound production seems to be shared for both EOs, whereas some seem to be specifically induced by each EO. These results support the hypothesis of different modes of action for each EO and further demonstrated the plant’s reaction to these EO injections. The differences between the two EO profiles may result from their specific interactions with the plant and, more precisely, with the plasma membrane. Recently, molecular techniques of dynamic interaction were applied to study the interaction between a biomimetic membrane with monoterpene (citronellal and citronellol) and with cinnamaldehyde (phenylpropanoids). Briefly, the *in silico* insertion model predicted different behaviours between the two classes (monoterpenes and phenylpropanoids), which are the stable interactions with plant lipids for monoterpene, whilst *trans*-cinnamaldehydes had no stable interaction with the membrane. These predictions were confirmed using *in vitro* biophysical assays (Lins et al., 2019).

Regarding the contained VOC profiles, green leaf volatiles (GLVs) generated by the lipoxygenase (LOX) pathway such as 2-hexenal constitute the major compounds. Due to the extraction protocol, this profile may not be interpreted as a potential pool for VOC emissions in the environment, as *de novo* synthesis could have occurred during incubation and trapping after grinding, especially for GLV. DHS is the most widely used sampling approach in the plant field because of its flexibility (sampled volume, trapping approaches and materials) (Bicchi et al., 2008). A high concentration factor was applied for the trace components under study. However, the analysis of these contained profiles may prove useful to further establish the metabolomic impact of EOs injection into apple trees. The presence of greater amounts of other aldehydes, such as nonanal, and the plant volatile hormone methyl salicylate reinforces the previously formulated hypothesis of resistance induction (Wenig et al., 2019). Other compounds emerged from the degradation of carotenoids, namely β-ionone and homocyclocitral (Dudareva et al., 2013).

Our work did not express foliar phytotoxicity. Chlorophyll fluorescence is a non-destructive and sensitive method that is widely used in eco-physiological studies to assess abiotic stress in plants. Indeed, perturbation in plant metabolism may decrease photosystem II (PSII) performance. However, local toxicity at the injection site cannot be excluded, as well as the mechanical damage that occurs due to the injection procedure (Doccola, 2012; Aćimović et al., 2016a). Furthermore, the specific mode of action of carvone can lead to microtubule depolarisation within cells. Lastly, unspecific generation of ROS has been frequently observed after EO application (Kaur et al., 2010; Sunohara et al., 2015; Dahiya et al., 2020). Carotenoids are amongst the first non-enzymatic antioxidants acting to protect photosystem II from photo-inhibition and ROS (Pospíšil, 2012). Therefore, the higher content of its degradation product in the leaf may be explained by such a phenomenon. Physiological disorder in phytohormones or ROS balances that may result in chronic and long-term toxicity from EO applications should be addressed before concluding a lack of harmful effects of the treatments.

In terms of agricultural application, trunk injection and EO applications are rarely used (Aćimović et al., 2020). This work was established as a proof of concept that the combination of both may be a suitable strategy to develop the biopesticidal potential of EOs whilst avoiding most of their drawbacks. However, we must highlight that more works in terms of reproducibility of results over different years, with other apple varieties, rootstock and efficiency on diverse pests are needed to establish the agronomic potential of such treatment. The absence of impact on apple quality or yield and on tree growth through long-term phytotoxicity should be established as well. Field trials should be performed to establish efficacy as a biopesticide and the lack of harmful effect to beneficial insects.

Plant VOCs are a promising tool, as they have numerous applications in agriculture, such as parasitoid attractant or through defence induction or priming, growth regulators and abiotic stress protectants (Brilli et al., 2019). Moreover, the use of natural substances that elicit systemic resistance has been proven to be a suitable strategy for pathogen management in orchards (Lateur, 2002). The possibility of combining EOs due to their biopesticidal properties with a new mode of application—trunk injection—was hereby demonstrated. Furthermore, the variations in the emitted and contained VOCs clearly demonstrate that young apple trees react to EO injection and that this reaction may be explored to design sustainable agricultural practices.

## Data Availability Statement

The original contributions presented in the study are included in the article/Supplementary Material, further inquiries can be directed to the corresponding author/s.

## Author Contributions

P-YW and CB: conceptualisation, methodology, formal analysis, and writing—original draft preparation. P-YW, CB, M-LF, GL, and TH: validation and writing—review and editing. M-LF: supervision. M-LF, GL, and TH: project administration and funding acquisition. All authors contributed to the article and approved the submitted version.

## Conflict of Interest

The authors declare that the research was conducted in the absence of any commercial or financial relationships that could be construed as a potential conflict of interest.

## Footnotes

1 www.metaboanalyst.ca

## References

[B1] Aćimović S. G. (2014). *Disease Management in Apples Using Trunk Injection Delivery of Plant Protective Compounds.* 10.13140/2.1.2252.3841 PhD Thesis, Michigan State University, Michigan.

[B2] Aćimović S. G. Cregg B. M. Sundin G. W. Wise J. C. (2016a). Comparison of drill- and needle-based tree injection technologies in healing of trunk injection ports on apple trees. *Urban For. Urban Green.* 19 151–157. 10.1016/j.ufug.2016.07.003

[B3] Aćimović S. G. Martin D. K. H. Turcotte R. M. Meredith C. L. Munck I. A. (2020). “Choosing an adequate pesticide delivery system for managing pathogens with difficult biologies: case studies on *Diplodia corticola*, *Venturia inaequalis* and *Erwinia amylovora*,” in *Plant Diseases - Current Threats and Management Trends*, ed. Topolovec-Pintaric S. (London: IntechOpen), 10.5772/intechopen.87956

[B4] Aćimović S. G. VanWoerkom A. H. Garavaglia T. Vandervoort C. Sundin G. W. Wise J. C. (2016b). Seasonal and cross-seasonal timing of fungicide trunk injections in apple trees to optimize management of apple scab. *Plant Dis.* 100 1606–1616. 10.1094/PDIS-09-15-1061-RE 30686216

[B5] Aćimović S. G. VanWoerkom A. H. Reeb P. D. Vandervoort C. Garavaglia T. Cregg B. M. (2014). Spatial and temporal distribution of trunk-injected imidacloprid in apple tree canopies. *Pest Manag. Sci.* 70 1751–1760. 10.1002/ps.3747 24481641

[B6] Aćimović S. G. Zeng Q. McGhee G. C. Sundin G. W. Wise J. C. (2015). Control of fire blight (*Erwinia amylovora*) on apple trees with trunk-injected plant resistance inducers and antibiotics and assessment of induction of pathogenesis-related protein genes. *Front. Plant Sci.* 6:16. 10.3389/fpls.2015.00016 25717330 PMC4323746

[B7] Alins G. Alegre S. Avilla J. (2017). Alternative to azadirachtin to control *Dysaphis plantaginea* Passerini (Hemiptera: Aphidae) in organic apple production. *Biol. Agric. Hortic.* 33 235–246. 10.1080/01448765.2017.1333454

[B8] Anderson M. J. (2017). “Permutational multivariate analysis of variance (PERMANOVA),” in *Wiley StatsRef: Statistics Reference Online*, ed. cf. Wiley (Hoboken, NJ: John Wiley & Sons, Ltd), 1–15. 10.1002/9781118445112.stat07841

[B9] Asakawa Y. Sekita M. Hashimoto T. (2018). Biotransformation of bicyclic sesqui- and diterpene 1,2-dials and their derivatives by the fungus, *Aspergillus Niger*. *Nat. Prod. Commun.* 13 923–932. 10.1177/1934578x1801300802

[B10] Bakkali F. Averbeck S. Averbeck D. Idaomar M. (2008). Biological effects of essential oils - A review. *Food Chem. Toxicol.* 46 446–475. 10.1016/j.fct.2007.09.106 17996351

[B11] Bicchi C. Cordero C. Liberto E. Sgorbini B. Rubiolo P. (2008). Headspace sampling of the volatile fraction of vegetable matrices. *J. Chromatogr. A* 1184, 220–233. 10.1016/j.chroma.2007.06.019 17624361

[B12] Bresson J. Bieker S. Riester L. Doll J. Zentgraf U. (2018). A guideline for leaf senescence analyses: from quantification to physiological and molecular investigations. *J. Exp. Bot.* 69 769–786. 10.1093/jxb/erx246 28992225

[B13] Brilli F. Loreto F. Baccelli I. (2019). Exploiting plant volatile organic compounds (VOCs) in agriculture to improve sustainable defense strategies and productivity of crops. *Front. Plant Sci.* 10:264. 10.3389/fpls.2019.00264 30941152 PMC6434774

[B14] Calfapietra C. Pallozzi E. Lusini I. Velikova V. Monson R. K. Niinemets Ü (2013). *Biology, Controls and Models of Tree Volatile Organic Compound Emissions.* Dordrecht: Springer Netherlands, 10.1007/978-94-007-6606-8

[B15] Campos E. V. R. Proença P. L. F. Oliveira J. L. Bakshi M. Abhilash P. C. Fraceto L. F. (2019). Use of botanical insecticides for sustainable agriculture: future perspectives. *Ecol. Indic.* 105 483–495. 10.1016/j.ecolind.2018.04.038

[B16] Casado D. Gemeno C. Avilla J. Riba M. (2006). Day-Night and phenological variation of apple tree volatiles and electroantennogram responses in *Cydia pomonella* (Lepidoptera: Tortricidae). *Environ. Entomol.* 35 258–267. 10.1603/0046-225X-35.2.258 33044624

[B17] Chizzali C. Beerhues L. (2012). Phytoalexins of the pyrinae: biphenyls and dibenzofurans. *Beilstein J. Org. Chem.* 8 613–620. 10.3762/bjoc.8.68 22563359 PMC3343287

[B18] Coslor C. C. Vandervoort C. Wise J. C. (2019). Insecticide dose and seasonal timing of trunk injection in apples influence efficacy and residues in nectar and plant parts. *Pest Manag. Sci.* 75 1453–1463. 10.1002/ps.5268 30450658

[B19] Dahiya S. Batish D. R. Singh H. P. (2020). *Pogostemon benghalensis* essential oil inhibited the weed growth via causing oxidative damage. *Rev. Bras. Bot.* 43 447–457. 10.1007/s40415-020-00613-8

[B20] Damalas C. A. Eleftherohorinos I. G. (2011). Pesticide Exposure, Safety Issues, and Risk Assessment Indicators. *Int. J. Environ. Res. Public Health* 8 1402–1419. 10.3390/ijerph8051402 21655127 PMC3108117

[B21] Damos P. Colomar L. A. E. Ioriatti C. (2015). Integrated fruit production and pest management in europe: the apple case study and how far we are from the original concept? *Insects* 6 626–657. 10.3390/insects6030626 26463407 PMC4598656

[B22] De Clerck C. Maso S. D. Parisi O. Dresen F. Zhiri A. Haissam Jijakli M. (2020). Screening of antifungal and antibacterial activity of 90 commercial essential oils against 10 pathogens of agronomical importance. *Foods* 9:1418. 10.3390/foods9101418 33036495 PMC7599922

[B23] Delory B. M. Delaplace P. du Jardin P. Fauconnier M. L. (2016). Barley (*Hordeum distichon* L.) roots synthesise volatile aldehydes with a strong age-dependent pattern and release (E)-non-2-enal and (E,Z)-nona-2,6-dienal after mechanical injury. *Plant Physiol. Biochem.* 104 134–145. 10.1016/j.plaphy.2016.03.028 27031425

[B24] Doccola J. Wild P. (2012). *Tree Injection as an Alternative Method of Insecticide Application.* Woburn, MA: Arborjet, Inc, 10.5772/29560

[B25] Doccola J. J. (2012). Treatment strategies using imidacloprid in Hemlock Woolly Adelgid (*Adelges tsugae* Annand) infested eastern Hemlock (*Tsuga canadensis* Carrière) trees. *Arboricul. Urban For.* 38 41–49.

[B26] Dudareva N. Klempien A. Muhlemann J. K. Kaplan I. (2013). Biosynthesis, function and metabolic engineering of plant volatile organic compounds. *New Phytol.* 198 16–32. 10.1111/nph.12145 23383981

[B27] Dugé De Bernonville T. Marolleau B. Staub J. Gaucher M. Brisset M. N. (2014). Using molecular tools to decipher the complex world of plant resistance inducers: an apple case study. *J. Agric. Food Chem.* 62 11403–11411. 10.1021/jf504221x 25372566

[B28] Durenne B. Blondel A. Druart P. Fauconnier M. L. (2018). A laboratory high-throughput glass chamber using dynamic headspace TD-GC/MS method for the analysis of whole *Brassica napus* L. plantlet volatiles under cadmium-related abiotic stress. *Phytochem. Anal.* 29 463–471. 10.1002/pca.2750 29460984 PMC6099401

[B29] European Chemical Agency (2020). *Homepage - ECHA.* Available online at: https://echa.europa.eu/home (accessed December 10, 2020).

[B30] Geiger F. Bengtsson J. Berendse F. Weisser W. W. Emmerson M. Morales M. B. (2010). Persistent negative effects of pesticides on biodiversity and biological control potential on European farmland. *Basic Appl. Ecol.* 11 97–105. 10.1016/j.baae.2009.12.001

[B31] Gershenzon J. Dudareva N. (2007). The function of terpene natural products in the natural world. *Nat. Chem. Biol.* 3 408–414. 10.1038/nchembio.2007.5 17576428

[B32] Giacomuzzi V. Cappellin L. Nones S. Khomenko I. Biasioli F. Knight A. L. (2017). Diel rhythms in the volatile emission of apple and grape foliage. *Phytochemistry* 138 104–115. 10.1016/j.phytochem.2017.03.001 28291597

[B33] Hare J. D. (2011). Ecological role of volatiles produced by plants in response to damage by herbivorous insects. *Annu. Rev. Entomol.* 56 161–180. 10.1146/annurev-ento-120709-144753 21133760

[B34] Holopainen J. K. Gershenzon J. (2010). Multiple stress factors and the emission of plant VOCs. *Trends Plant Sci.* 15 176–184. 10.1016/j.tplants.2010.01.006 20144557

[B35] Hüsnü Can Başer K. Buchbauer G. (2015). *Handbook of Essential Oils: Science, Technology, and Applications.* Boca Raton, FL: CRC Press.

[B36] Ibdah M. Chen Y. T. Wilkerson C. G. Pichersky E. (2009). An aldehyde oxidase in developing seeds of arabidopsis converts benzaldehyde to benzoic acid. *Plant Physiol.* 150 416–423. 10.1104/pp.109.135848 19297586 PMC2675751

[B37] Ikbal C. Pavela R. (2019). Essential oils as active ingredients of botanical insecticides against aphids. *J. Pest Sci.* 92 971–986. 10.1007/s10340-019-01089-6

[B38] Isman M. B. (2020). Commercial development of plant essential oils and their constituents as active ingredients in bioinsecticides. *Phytochem. Rev.* 19 235–241. 10.1007/s11101-019-09653-9

[B39] Isman M. B. Miresmailli S. Machial C. (2011). Commercial opportunities for pesticides based on plant essential oils in agriculture, industry and consumer products. *Phytochem. Rev.* 10 197–204. 10.1007/s11101-010-9170-4

[B40] Jamar L. Cavelier M. Lateur M. (2010). Primary scab control using a “during-infection” spray timing and the effect on fruit quality and yield in organic apple production. *Biotechnol. Agron. Soc. Environ.* 14 423–439.

[B41] Kalaji H. M. Jajoo A. Oukarroum A. Brestic M. Zivcak M. Samborska I. A. (2016). Chlorophyll a fluorescence as a tool to monitor physiological status of plants under abiotic stress conditions. *Acta Physiol. Plant* 38:102. 10.1007/s11738-016-2113-y

[B42] Kaur S. Singh H. P. Mittal S. Batish D. R. Kohli R. K. (2010). Phytotoxic effects of volatile oil from *Artemisia scoparia* against weeds and its possible use as a bioherbicide. *Ind. Crops Prod.* 32 54–61. 10.1016/j.indcrop.2010.03.007

[B43] Kellerhals M. Szalatnay D. Hunziker K. Duffy B. Nybom H. Ahmadi-Afzadi M. (2012). European pome fruit genetic resources evaluated for disease resistance. *Trees Struct. Funct.* 26 179–189. 10.1007/s00468-011-0660-9

[B44] Koul O. Walia S. Dhaliwal G. (2008). Essential oils as green pesticides: potential and constraints. *Biopestic. Int.* 4 63–84.

[B45] Lateur M. (2002). Perspectives de lutte contre les maladies des arbres fruitiers à pépins au moyen de substances naturelles inductrices d’une résistance systémique. *BASE* 6 67–77.

[B46] Lee J. E. Seo S. M. Huh M. J. Lee S. C. Park I. K. (2020). Reactive oxygen species mediated-antifungal activity of cinnamon bark (*Cinnamomum verum*) and lemongrass (*Cymbopogon citratus*) essential oils and their constituents against two phytopathogenic fungi. *Pestic. Biochem. Physiol.* 168:104644. 10.1016/j.pestbp.2020.104644 32711777

[B47] Liberto E. Bicchi C. Cagliero C. Cordero C. Rubiolo P. Sgorbini B. (2020). “Headspace sampling: an ‘evergreen’ method in constant evolution to characterize food flavors through their volatile fraction,” in *Food Chemistry, Function and Analysis*, eds Williamson G. Marangoni A. G. Bonwick G. A. Birch C. S. (Burlington House: Royal Society of Chemistry), 3–37. 10.1039/9781788015752-00001

[B48] Lins L. Dal Maso S. Foncoux B. Kamili A. Laurin Y. Genva M. (2019). Insights into the relationships between herbicide activities, molecular structure and membrane interaction of cinnamon and citronella essential oils components. *Int. J. Mol. Sci.* 20:4007. 10.3390/ijms20164007 31426453 PMC6720526

[B49] Lopez-Reyes J. G. Spadaro D. Gullino M. L. Garibaldi A. (2010). Efficacy of plant essential oils on postharvest control of rot caused by fungi on four cultivars of apples *in vivo*. *Flavour Fragr. J.* 25 171–177. 10.1002/ffj.1989

[B50] Maes C. Bouquillon S. Fauconnier M. L. (2019). Encapsulation of essential oils for the development of biosourced pesticides with controlled release: a review. *Molecules* 24:2539. 10.3390/molecules24142539 31336803 PMC6680563

[B51] Marolleau B. Gaucher M. Heintz C. Degrave A. Warneys R. Orain G. (2017). When a plant resistance inducer leaves the lab for the field: integrating ASM into routine apple protection practices. *Front. Plant Sci.* 8:1938. 10.3389/fpls.2017.01938 29255473 PMC5723009

[B52] Matsui K. Sugimoto K. Mano J. Ozawa R. Takabayashi J. (2012). Differential metabolisms of green leaf volatiles in injured and intact parts of a wounded leaf meet distinct ecophysiological requirements. *PLoS One* 7:e36433. 10.1371/journal.pone.0036433 22558466 PMC3340338

[B53] Mbili N. C. Opara U. L. Lennox C. L. Vries F. A. (2017). Citrus and lemongrass essential oils inhibit *Botrytis cinerea* on ‘Golden Delicious’, ‘Pink Lady’ and ‘Granny Smith’ apples. *J. Plant Dis. Prot.* 124 499–511. 10.1007/s41348-017-0121-9

[B54] Miller S. S. Tworkoski T. (2010). Blossom thinning in apple and peach with an essential oil. *HortScience* 45 1218–1225. 10.21273/hortsci.45.8.1218

[B55] Montecchio L. (2013). A venturi effect can help cure our trees. *J. Vis. Exp.* 2013:51199. 10.3791/51199 24121874 PMC3938323

[B56] Moretti M. D. L. Sanna-Passino G. Demontis S. Bazzoni E. (2002). Essential oil formulations useful as a new tool for insect pest control. *AAPS PharmSci. Tech.* 3 64–74. 10.1208/pt030213 12916950 PMC2750315

[B57] Muchembled J. Deweer C. Sahmer K. Halama P. (2018). Antifungal activity of essential oils on two *Venturia inaequalis* strains with different sensitivities to tebuconazole. *Environ. Sci. Pollut. Res.* 25 29921–29928. 10.1007/s11356-017-0507-z 29098578

[B58] Nea F. Tanoh E. A. Wognin E. L. Kenne Kemene T. Genva M. Saive M. (2019). A new chemotype of *Lantana rhodesiensis* Moldenke essential oil from Côte d’Ivoire: chemical composition and biological activities. *Ind. Crops Prod.* 141:111766. 10.1016/j.indcrop.2019.111766

[B59] Pang Z. Chong J. Li S. Xia J. (2020). MetaboAnalystR 3.0: toward an optimized workflow for global metabolomics. *Metabolites* 10:186. 10.3390/metabo10050186 32392884 PMC7281575

[B60] Paré P. W. Tumlinson J. H. (1999). Plant volatiles as a defense against insect herbivores. *Plant Physiol.* 121 325–331. 10.1104/pp.121.2.325 10517823 PMC1539229

[B61] Patil I. (2018). *{ggstatsplot}: “ggplot2” Based Plots with Statistical Details}.* Available online at: https://dmetar.protectlab.org/authors.html (accessed December 9, 2020).

[B62] Percival G. C. Boyle S. (2005). Evaluation of microcapsule trunk injections for the control of apple scab and powdery mildew. *Ann. Appl. Biol.* 147 119–127. 10.1111/j.1744-7348.2005.00019.x

[B63] Perina F. J. de Andrade C. C. L. Moreira S. I. Nery E. M. Ogoshi C. Alves E. (2019). *Cinnamomun zeylanicum* oil and trans-cinnamaldehyde against Alternaria brown spot in tangerine: direct effects and induced resistance. *Phytoparasitica* 47 575–589. 10.1007/s12600-019-00754-x

[B64] Pospíšil P. (2012). Molecular mechanisms of production and scavenging of reactive oxygen species by photosystem II. *Biochim. Biophys. Acta Bioenerg.* 1817 218–231. 10.1016/j.bbabio.2011.05.017 21641332

[B65] Rehman R. Hanif M. A. Mushtaq Z. Al-Sadi A. M. (2016). Biosynthesis of essential oils in aromatic plants: a review. *Food Rev. Int.* 32 117–160. 10.1080/87559129.2015.1057841

[B66] Riedlmeier M. Ghirardo A. Wenig M. Knappe C. Koch K. Georgii E. (2017). Monoterpenes support systemic acquired resistance within and between plants. *Plant Cell* 29 1440–1459. 10.1105/tpc.16.00898 28536145 PMC5502447

[B67] Rivas F. Parra A. Martinez A. Garcia-Granados A. (2013). Enzymatic glycosylation of terpenoids. *Phytochem. Rev.* 12 327–339. 10.1007/s11101-013-9301-9

[B68] Rousselin A. Bevacqua D. Sauge M. H. Lescourret F. Mody K. Jordan M. O. (2017). Harnessing the aphid life cycle to reduce insecticide reliance in apple and peach orchards. A review. *Agron. Sustain. Dev.* 37:38. 10.1007/s13593-017-0444-8

[B69] Saini S. S. Teotia D. Gaid M. Sircar D. (2019). A new enzymatic activity from elicitor-treated pear cell cultures converting *trans* -cinnamic acid to benzaldehyde. *Physiol. Plant.* 167 64–74. 10.1111/ppl.12871 30417393

[B70] Singh P. Pandey A. K. (2018). Prospective of essential oils of the genus mentha as biopesticides: a review. *Front. Plant Sci.* 9:1295. 10.3389/fpls.2018.01295 30250476 PMC6139362

[B71] Snoussi M. Noumi E. Trabelsi N. Flamini G. Papetti A. De Feo V. (2015). Mentha spicata essential oil: chemical composition, antioxidant and antibacterial activities against planktonic and biofilm cultures of *Vibrio spp.* Strains. *Molecules* 20 14402–14424. 10.3390/molecules200814402 26262604 PMC6332415

[B72] Souleyre E. J. F. Bowen J. K. Matich A. J. Tomes S. Chen X. Hunt M. B. (2019). Genetic control of α-farnesene production in apple fruit and its role in fungal pathogenesis. *Plant J.* 100 1148–1162. 10.1111/tpj.14504 31436867

[B73] Souza B. Lundgren J. Rodriguez-Saona C. (2017). From laboratory to field: electro-antennographic and behavioral responsiveness of two insect predators to methyl salicylate. *Chemoecology* 27 51–63. 10.1007/s00049-017-0230-8

[B74] Stewart-Jones A. Poppy G. M. (2006). Comparison of glass vessels and plastic bags for enclosing living plant parts for headspace analysis. *J. Chem. Ecol.* 32 845–864. 10.1007/s10886-006-9039-6 16718573

[B75] Suckling D. M. Twidle A. M. Gibb A. R. Manning L. M. Mitchell V. J. Sullivan T. E. S. (2012). Volatiles from apple trees infested with light brown apple moth larvae attract the parasitoid *Dolichogenidia tasmanica*. *J. Agric. Food Chem.* 60 9562–9566. 10.1021/jf302874g 22950817

[B76] Sunohara Y. Baba Y. Matsuyama S. Fujimura K. Matsumoto H. (2015). Screening and identification of phytotoxic volatile compounds in medicinal plants and characterizations of a selected compound, eucarvone. *Protoplasma* 252 1047–1059. 10.1007/s00709-014-0739-4 25534256

[B77] Synowiec A. Możdżeń K. Krajewska A. Landi M. Araniti F. (2019). *Carum carvi* L. essential oil: a promising candidate for botanical herbicide against *Echinochloa crus-galli* (L.) P. Beauv. in maize cultivation. *Ind. Crops Prod.* 140:111652. 10.1016/j.indcrop.2019.111652

[B78] Synowiec A. Mozdzeń K. Skoczowski A. (2015). Early physiological response of broccoli leaf to foliar application of clove oil and its main constituents. *Ind. Crops Prod.* 74 523–529. 10.1016/j.indcrop.2015.05.069

[B79] Tanoh E. A. Boué G. B. Nea F. Genva M. Wognin E. L. Ledoux A. (2020). Seasonal effect on the chemical composition, insecticidal properties and other biological activities of *zanthoxylum leprieurii* guill. & perr. essential oils. *Foods* 9:550. 10.3390/foods9050550 32369948 PMC7278710

[B80] Teotia D. Gaid M. Saini S. S. Verma A. Yennamalli R. M. Khare S. P. (2019). Cinnamate-CoA ligase is involved in biosynthesis of benzoate-derived biphenyl phytoalexin in *Malus* × *domestica* ‘Golden Delicious’ cell cultures. *Plant J.* 100 1176–1192. 10.1111/tpj.14506 31437324

[B81] Trowbridge A. M. Stoy P. C. (2013). “BVOC-mediated plant-herbivore interactions,” in *Biology, Controls and Models of Tree Volatile Organic Compound Emissions*, ed. Russell K. M. (Dordrecht: Springer Netherlands), 21–46. 10.1007/978-94-007-6606-8_2

[B82] Vallat A. Gu H. Dorn S. (2005). How rainfall, relative humidity and temperature influence volatile emissions from apple trees in situ. *Phytochemistry* 66 1540–1550. 10.1016/j.phytochem.2005.04.038 15949824

[B83] Warneys R. Gaucher M. Robert P. Aligon S. Anton S. Aubourg S. (2018). Acibenzolar-s-methyl reprograms apple transcriptome toward resistance to rosy apple aphid. *Front. Plant Sci.* 9:1795. 10.3389/fpls.2018.01795 30619387 PMC6299034

[B84] Wenig M. Ghirardo A. Sales J. H. Pabst E. S. Breitenbach H. H. Antritter F. (2019). Systemic acquired resistance networks amplify airborne defense cues. *Nat. Commun.* 10:3813. 10.1038/s41467-019-11798-2 31444353 PMC6707303

[B85] Werrie P.-Y. Durenne B. Delaplace P. Fauconnier M.-L. (2020). Phytotoxicity of essential oils: opportunities and constraints for the development of biopesticides. A Review. *Foods* 9:1291. 10.3390/foods9091291 32937933 PMC7554882

[B86] Wise J. C. VanWoerkom A. H. Acimovic S. G. Sundin G. W. Cregg B. M. Vandervoort C. (2014). Trunk injection: an alternative technique for pesticide delivery in apples. *Crop Prot.* 65 173–185. 10.1016/j.cropro.2014.05.017

[B87] Zeng L. Wang X. Kang M. Dong F. Yang Z. (2017). Regulation of the rhythmic emission of plant volatiles by the circadian clock. *Int. J. Mol. Sci.* 18:2408. 10.3390/ijms18112408 29137171 PMC5713376

[B88] Zhang C. Fan L. Fan S. Wang J. Luo T. Tang Y. (2019). *Cinnamomum cassia* Presl: a review of its traditional uses, phytochemistry, pharmacology and toxicology. *Molecules* 24:3473. 10.3390/molecules24193473 31557828 PMC6804248

