# Supplementary material for: Biopesticide Trunk Injection Into Apple Trees: A Proof of Concept for the Systemic Movement of Mint and Cinnamon Essential Oils
Source: Front Plant Sci. 2021 Apr 9;12:650132. doi: 10.3389/fpls.2021.650132 (PMC8063119; doi:10.3389/fpls.2021.650132)
Supplement: Supplementary file 1 [file Data_Sheet_1.docx]

Supplementary Material

**Supplementary table S1.** Cinamomun cassia essential oil composition analysis.

| Name | Score (Lib) | CAS | Relative area (%) |
| --- | --- | --- | --- |
| Cinnamaldehyde, (E)- | 98 | 104-55-2 | 91.22 |
| o-Methoxycinnamaldehyde | 97.06 | 1504-74-1 | 1.98 |
| Benzaldehyde | 90.59 | 100-52-7 | 0.75 |
| Benzenepropanal | 93.23 | 104-53-0 | 0.60 |
| Copaene | 91.68 | 3856-25-5 | 0.55 |
| Benzaldehyde, 2-methoxy- | 93.18 | 135-02-4 | 0.39 |
| Butylated Hydroxytoluene | 89.6 | 128-37-0 | 0.39 |
| Acetic acid, cinnamyl ester | 95.36 | 103-54-8 | 2.30 |
| Benzaldehyde, 2-hydroxy- | 85.01 | 90-02-8 | 0.24 |
| Caryophyllene | 89.78 | 87-44-5 | 0.23 |
| Acetic acid, 2-phenylethyl ester | 93.29 | 103-45-7 | 0.21 |
| Benzofuran, 2-methyl- | 87.02 | 4265-25-2 | 0.20 |
| Naphthalene-hexahydro-4,7-dimethyl-1-(1-methylethyl)-, (1S-cis)- | 84.6 | 483-76-1 | 0.19 |
| Phenylethyl Alcohol | 85.89 | 60-12-8 | 0.18 |
| (1S,4aR,8aS)-1-Isopropyl-7-methyl-4-methylene-octahydronaphthalene | 82.39 | 6980-46-7 | 0.13 |
| Total |  |  | 99.56 |

**Supplementary table S2.** Mentha spicata essential oil composition analysis.

| Name | Score (Lib) | CAS | Relative area (%) |
| --- | --- | --- | --- |
| Carvone | 93.77 | 99-49-0 | 57.78 |
| D-Limonene | 95.74 | 138-86-3 | 25.28 |
| Dihydrocarvone | 93.55 | 5524-05-0 | 2.06 |
| (-)-β-Bourbonene | 94.35 | 5208-59-3 | 1.72 |
| Caryophyllene | 96.81 | 87-44-5 | 1.57 |
| β-Pinene | 92.87 | 127-91-3 | 1.34 |
| β-Myrcene | 94.27 | 123-35-3 | 1.33 |
| Unknown terpene |  |  | 0.86 |
| Terpinen-4-ol | 93.68 | 562-74-3 | 0.72 |
| Germacrene D | 95.26 | 23986-74-5 | 0.71 |
| Piperitone | 85.22 | 89-81-6 | 0.6 |
| Levomenthol | 97.34 | 2216-51-5 | 0.52 |
| (E)-β-Farnesene | 94.57 | 18794-84-8 | 0.41 |
| Butylated Hydroxytoluene | 90.77 | 128-37-0 | 0.31 |
| Dihydrocarvyl acetate | 95.79 | 20777-49-5 | 0.28 |
| 4-Thujanol | 88.97 | 546-79-2 | 0.27 |
| γ-Terpinene | 92.26 | 99-85-4 | 0.25 |
| α-Terpineol | 93.61 | 98-55-5 | 0.25 |
| unidentified |  |  | 0.25 |
| cis-sabinene | 92.58 | 3387-41-5 | 0.23 |
| o-Cymene | 91.21 | 527-84-4 | 0.23 |
| Carvyl acetate (Z) | 94.47 | 1205-42-1 | 0.22 |
| Acetic acid, hexyl ester | 89.91 | 142-92-7 | 0.21 |
| trans-Carveol | 89.07 | 1197-07-5 | 0.21 |
| α-Bisabolene | 88.31 | 17627-44-0 | 0.2 |
| Isogermacrene D | 90.5 | 317819-80-0 | 0.19 |
| Naphthalene, -hexahydro-4,7-dimethyl-1-(1-methylethyl) | 90.89 | 483-76-1 | 0.16 |
| α-Terpinolene | 81.96 | 586-62-9 | 0.15 |
| Dihydroedulan | 81.8 | 41678-32-4 | 0.15 |
| (E)-β-Elemene | 91.48 | 515-13-9 | 0.14 |
| Unidentified sesquiterpene |  |  | 0.14 |
| Isomenthone | 90.73 | 491-07-6 | 0.13 |
| Caryophyllene oxide | 83.74 | 1139-30-6 | 0.13 |
| (+)-4-Carene | 83.46 | 29050-33-7 | 0.12 |
| 3-Octanol, acetate | 88.16 | 4864-61-3 | 0.12 |
| δ-Terpineol | 84.64 | 98-55-5 | 0.1 |
| Limonene oxide, trans- | 82.77 | 4959-35-7 | 0.08 |
| γ-Muurolene | 84.54 | 30021-74-0 | 0.05 |
| 1,3,6-Heptatriene, 2,5,6-trimethyl- | 81.89 | 42123-66-0 | 0.04 |
| Linalool | 82.17 | 78-70-6 | 0.02 |
| Total |  |  | 99.53 |

**Supplementary table S3.** Untargeted VOC emissions profiles (TDU-GC-MS): detailed composition of headspace emissions profiles of *Malus domestica* tree belonging to the alkanes, alkenes, alcohol, aldehydes, aliphatic and aromatic esters, furanes, homoterpenes, ketones, monoterpenes, sesquiterpenes and terpenoids. Compounds with Asterisks indicate significant differences after one-way ANOVA and Different letters indicate significant differences based on post hoc Tukey’s HSD test.

| **Family** | **Name** | **Calculated RI** | **Literature RI** | **Blank (n=11)** | **Cinnamon (n=5)** | **Mint (n=8)** | **Control (n=7)** |
| --- | --- | --- | --- | --- | --- | --- | --- |
| Alcohol/Phenol | 1-Octen-3-ol | 963.3 | 962 | 0.33±0.14 | 0.13±0.08 | 0.21±0.14 | 0.11±0.06 |
|  | 2-ethyl-1-Hexanol | 1772.8 | 1790 | 0.15±0.09 | 0.16±0.11 | 0.16±0.11 | 0.11±0.06 |
|  | 3-Hexen-1-ol, (Z)- | 839.3 | 856.6 | 0.67±0.3 | 0.51±0.23 | 0.11±0.04 | 0.36±0.14 |
|  | l-Menthol | 1153.7 | 1150.0 | 0.12±0.06 | n.d. | n.d. | 0.05±0.02 |
|  | 1-Hexanol | 853.2 | 869.7 | 0.21±0.06 | 0.74±0.33 | 0.12±0.04 | n.d. |
|  | 1-Octanol | 1219.1 | 1218.3 | 0.22±0.07 | 0.24±0.11 | 0.18±0.09 | n.d. |
| Aldehydes | 2-Decenal, (E)- | 1243.2 | 1263.4 | 0.11±0.03 | 0.07±0.03 | 0.07±0.02 | n.d. |
|  | 4-Methylhexen-2-enal* | 1013.1 | 1011.5 | 0.1±0.07^a^ | n.d. | n.d. | 0.07±0.04^ab^ |
|  | Decanal | 1186 | 1205.4 | 0.18±0.14 | 0.43±0.34 | 0.37±0.33 | 0.15±0.11 |
|  | Dodecanal | 1385.8 | 1408.1 | n.d. | 0.05±0.02 | 0.1±0.04 | n.d. |
|  | Nonanal | 1086.1 | 1103.3 | 0.29±0.33 | 0.93±1.23 | 0.74±0.68 | 0.19±0.22 |
|  | Undecanal | 1285.7 | 1306.5 | 0.06±0.02 | 0.08±0.04 | 0.1±0.05 | 0.05±0.02 |
| Aliphatic esters | 3-Hexen-1-ol, acetate, (Z)- | 993.3 | 1011.5 | 3.27±1.34 | 1.44±1.22 | 0.48±0.25 | 2.33±0.88 |
| Alkanes | 6-Methyldodecane | 1196.1 | 1250 | 0.06±0.02 | n.d. | 0.06±0.03 | 0.05±0.03 |
|  | Decane, 2,3,5,8-tetramethyl- | 656.7 | 720 | 0.08±0.04 | 0.09±0.04 | 0.08±0.05 | 0.06±0.03 |
|  | Dodecane, 2,6,11-trimethyl- | 686.7 | 776 | 0.14±0.08 | 0.16±0.1 | 0.13±0.08 | 0.11±0.03 |
|  | Nonane, 4,5-dimethyl-* | 1205.7 | 1205.4 | n.d. | 0.08±0.04^ab^ | 0.06±0.03^ab^ | 0.06±0.02^a^ |
|  | Dodecane | 1180.3 | 1200 | 0.16±0.1 | 0.14±0.09 | 0.19±0.1 | 0.13±0.04 |
|  | Phytane | 1782.9 | 1790 | 0.06±0.02 | n.d. | 0.06±0.03 | n.d. |
|  | Tetradecane | 1377 | 1400 | 0.21±0.13 | 0.14±0.06 | 0.19±0.12 | 0.16±0.03 |
|  | Tridecane | 1279 | 1300 | 0.25±0.15 | 0.2±0.12 | 0.29±0.16 | 0.22±0.05 |
|  | Undecane | 1082 | 1100 | 0.17±0.13 | 0.17±0.13 | 0.16±0.09 | 0.08±0.05 |
|  | Unidentified alkane RI 1477* | 1477 | - | 0.12±0.07^a^ | 0.1±0.06^ab^ | 0.12±0.07^ab^ | n.d |
|  | Unidentified RI 1174 | 1174 | - | n.d. | 0.11±0.05 | 0±0 | 0.04±0.01 |
|  | Unidentified alkane RI 1675 | 1675 | - | 0.08±0.03 | 0.09±0.04 | 0.07±0.04 | n.d. |
|  | 3-Ethyl-2,6,10-Trimethylundecane | 1441.6 | - | 0.09±0.05 | 0.13±0.08 | 0.09±0.05 | n.d. |
|  | 2,4-Dimethyldecane | 1040.2 | 1106 | 0.12±0.09 | 0.1±0.05 | 0.06±0.04 | 0.08±0.04 |
|  | Dodecane, 4,6-dimethyl- | 1360 | - | n.d. | n.d. | n.d. | 0.05±0.03 |
|  | Pristane | 1681.2 | 1684 | 0.06±0.02 | n.d. | 0.07±0.04 | n.d. |
| Alkenes | 7-Tetradecene | 1371.3 | 1374 | 0.07±0.04 | n.d. | 0.08±0.05 | n.d. |
| Aromatic esters | 3-Hexen-1-ol, benzoate, (Z)- | 1550.1 | 1550 | n.d. | 0.13±0.06 | 0.31±0.11 | n.d. |
|  | Methyl salicylate | 1177.4 | 1192.9 | n.d. | n.d. | 0.66±0.41 | n.d. |
| Esters | Isobornyl propionate | 1169.9 | 1171.3 | 0.11±0.07 | 0.2±0.13 | 0.13±0.05 | n.d. |
| Furanes | Trans-Linalool oxide | 1070.6 | 1071 | 0.1±0.06 | 0.21±0.16 | 0.18±0.1 | 0.06±0.03 |
| Homoterpenes | TMTT* | 1557.7 | 1566 | n.d. | 0.21±0.12^a^ | 0.2±0.16 ^a^ | 0.16±0.08 ^ab^ |
|  | DMNT | 1098.3 | 1105 | 0.98±0.54 | 0.79±0.52 | 2.58±2.53 | 0.46±0.28 |
| Ketones | 2-Undecanone | 1276.5 | 1294 | 0.06±0.03 | 0.1±0.04 | 0.07±0.04 | n.d. |
|  | trimethyl-2-Pentadecanone | 1833 | 1842 | 0.09±0.06 | 0.12±0.05 | 0.1±0.04 | 0.04±0.02 |
| Monoterpenes | Geranylacetone | 961.9 | NA | 0.07±0.02 | 0.18±0.08 | 0.14±0.06 | n.d. |
|  | l-Menthone | 1136.3 | 1136 | 0.04±0.02 | n.d. | 0.07±0.03 | n.d. |
|  | Dihydroactinidiolide | 1046.4 | 1011.3 | 0.08±0.02 | n.d. | 0.11±0.04 | n.d. |
|  | α-Bergamotene* | 987.9 | 977.7 | n.d. | 0.13±0.07^a^ | 0.14±0.08^a^ | 0.08±0.04^ab^ |
|  | β-Ocimene | 1033.7 | 1037.8 | n.d. | 0.07±0.03 | 0.36±0.33 | n.d |
| Sesquiterpenes | α-Farnesene* | 1484.2 | 1490.9 | 0.65±0.72^b^ | 6.27±1.81^a^ | 2±0.92^b^ | 1.56±2.23^b^ |
|  | α-Muurrolene | 1494.9 | 1498.3 | n.d. | 0.3±0.13 | 0.2±0.09 | n.d. |
|  | Caryophyllene* | 1398 | 1406.5 | 0.16±0.13^b^ | 0.45±0.21^ab^ | 0.67±0.44^a^ | 0.1±0.03^b^ |
|  | D-Cadinene | 1503.7 | 1523.2 | n.d. | 0.31±0.14 | 0.26±0.13 | n.d. |
|  | Germacrene D* | 1459.3 | 1480.6 | 0.33±0.26^b^ | 1.18±0.67^a^ | 0.73±0.39^ab^ | 0.35±0.21^b^ |
|  | γ-Muurolene | 1457.9 | 1476.2 | n.d. | 0.1±0.04 | 0.08±0.04 | n.d. |
| Terpenoids | D-Carvone* | 1178.7 | 1242 | n.d. | n.d. | 0.08±0.05^a^ | n.d |
|  | Linalool | 1084.6 | 1099 | n.d. | 0.45±0.2 | 0.3±0.19 | n.d |
|  | Terpinen-4-ol | 1159.6 | 1177.1 | n.d. | 0.14±0.06 | n.d. | n.d |
|  | Dihydrocarvone | 1179.2 | 1201.4 | n.d. | n.d. | 0.17±0.06 | n.d. |
| Unknown | Unidentified RI 1367 | 1367 | - | n.d. | n.d. | 0.07±0.03 | 0±0 |
|  | Unidentified RI 1273 | 1273 | - | n.d. | n.d. | 0.08±0.05 | 0.04±0.01 |
|  | Unidentified RI 1686 | 1686 | - | n.d. | n.d. | 0.08±0.03 | n.d. |

**Supplementary table S4.** Untargetted VOCs Contained (DHS-GC-MS): Detailed composition of VOCs contained in leaves of *Malus domestica*. These compounds belong to the alcohols, aldehydes, alkadienes, alkanes, aromatic and aliphatic esters, fatty acid esters, homoterpenes, ketones.

| **Family** | **Name** | **Calculated RI** | **Literature RI** | **Blank (n=15)** | **Cinnamon (n=15)** | **Mint (n=14)** | **Control (n=14)** |
| --- | --- | --- | --- | --- | --- | --- | --- |
| Alcohol/  Phenol | Terphenyl-2-ol * | 2213.9 | 2275 | 1.94±0.5^b^ | 0.91±0.43^b^ | 8.71±6.48^a^ | n.d. |
|  | 1-Octanol* | 1071.9 | 1272.1 | 1.66±0.43^b^ | 17.83±8.47^a^ | 9.14±3.97^b^ | n.d. |
|  | 1-Penten-3-ol* | 679 | 675 | 31.48±25.9^b^ | 333.67±284.47^a^ | 277.31±220.6^a^ | 19.38±8.17^b^ |
|  | 2,4-Hexadien-1-ol | 817.4 | 882 | n.d. | n.d. | 54.96±0 | 41.19±14.87 |
|  | 2-Octen-1-ol, (Z)- | 1052.6 | 1039 | n.d. | 3.26±1.11 | n.d. | n.d. |
|  | 2-Penten-1-ol, (Z)-* | 669.5 | 771.2 | 68.14±66.76^b^ | 333.22±274.02^a^ | 354±187.52^a^ | 47.82±67.9^b^ |
| Aldehydes | 2,4-Heptadienal* | 1013.1 | 1011.5 | 15.02±19.43^b^ | 67.57±24.21^a^ | 68.58±23.82^a^ | 34.63±19.95^b^ |
|  | 2,4-Hexadienal* | 913.6 | 913.2 | 73.37±99.68^b^ | 212.39±198.4^a^ | 47.29±44.9^b^ | 48.26±56.87^b^ |
|  | 2-Decenal, (E)-* | 1261.9 | 1263.4 | n.d. | 13.23±13.19^a^ | 9.3±7.01^b^ | 4.04±4.52^b^ |
|  | 2-Heptenal, (E)-* | 958.4 | 960.5 | 8.33±7.61^b^ | 42.5±29.31^a^ | 14.19±8.42^b^ | 18.5±7.86^b^ |
|  | 2-Hexenal, (E)-* | 796.3 | 853 | 1350.58±833.5^b^ | 3816.23±1634.35^a^ | 2384.14±1393.71^ab^ | 1145.79±1223.41^b^ |
|  | 2-Nonenal, (E)- * | 1160.4 | 1162.2 | n.d. | 2.4±1.77^a^ | 0.85±0^b^ | n.d. |
|  | 2-Octenal, (E)- | 1058.9 | 1060.2 | n.d. | 4.96±2.38 | 3.05±0.53 | n.d. |
|  | 2-Pentenal, (E)- | 656.7 | 720 | 1.47±0 | 60.03±82.45 | 6.62±2.29 | n.d. |
|  | Benzaldehyde* | 961.9 | 949 | 10.93±12.26^b^ | 22.69±17.12^b^ | 46,22±42,69^a^ | 12.64±11.23^b^ |
|  | Benzene-acetaldehyde* | 1044 | 1039 | 3.7±1.67^b^ | 7.89±4.73^a^ | n.d. | 0.9±0.47^b^ |
|  | Decanal* | 1205.7 | 1205.4 | 1.21±0.41^b^ | 4.45±2.95^ab^ | 5.78±7.72^a^ | 2.42±1.47^ab^ |
|  | Heptanal | 903.5 | 902 | 3.94±2.95 | 25.64±20.81 | 14.81±11.42 | 5.32±9.12 |
|  | Hexanal* | 734.2 | 799.9 | 278.56±348.69^b^ | 889.75±450.14^a^ | 299.17±205.76^b^ | 163.69±372.1^b^ |
|  | Nonanal* | 1104.8 | 1103.3 | 8.4±5.82^b^ | 51.19±34.08^a^ | 40.83±31.12^a^ | 11.17±13.04^b^ |
|  | Octanal* | 1004.1 | 1002.8 | 2.93±2.17^b^ | 13.66±10.98^a^ | 17.94±7.86^ab^ | 3.75±3.43^ab^ |
| Alkadiene | 3-Ethyl-1,5-octadiene* | 941.2 | 939 | n.d. | 2.53±1.19^ab^ | 8.65±9.13^a^ | n.d. |
| Alkanes | Decane, 2,3,6-trimethyl- | 1069.3 | 1466 | n.d. | 18.8±13.23 | 1.93±0 | n.d. |
|  | Dodecane | 1199.9 | 1200 | 1.03±0.45 | 1.33±0.69 | 2.63±2.11 | 1.02±0.66 |
|  | Heptadecane | 1699.1 | 1700 | n.d. | 1.56±0.45 | 1.55±0.95 | n.d. |
|  | Heptane, 2,2,4,6,6-pentamethyl- | 1010.1 | 1003 | n.d. | 8.7±14.54 | 2.96±1.94 | n.d. |
|  | Hexadecane | 1599 | 1600 | n.d. | 1.41±0.52 | 2.3±1.61 | n.d. |
|  | Nonane, 3-methyl- | 1051.3 | 970 | n.d. | 6.48±6.05 | 3.05±1.02 | n.d. |
|  | Octadecane | 1760.5 | 1800 | n.d. | 1.28±0.24 | 1.45±1.11 | n.d. |
|  | Tetradecane | 1399.5 | 1400 | 0.84±0 | 1.44±0.28 | 4.03±2.12 | n.d. |
| Aromatic esters | 1-ethyl-cyclohexene | 998.5 | 1011.5 | n.d. | 4.59±1.51 | 10.85±18.18 | n.d. |
|  | Methyl salicylate* | 1191.4 | 1192.9 | 3.82±2.78^b^ | 32.8±39.31^a^ | 41.82±32.97^a^ | 20.93±21.29^ab^ |
|  | 1,3,5-Trimethylbenzene | 990.7 | 996 | n.d. | n.d. | 9.76±9.5 | n.d. |
|  | 3-Hexen-1-ol benzoate | 1569.2 | 1569.5 | 1.6±0 | 15.28±21.95 | 5.29±5.1 | n.d. |
|  | Benzoic acid, ethyl ester | 1169.9 | 1171.3 | n.d. | 3.28±0 | 11.73±13.32 | n.d. |
| Aliphatic esters | 3-Hexen-1-ol, acetate, (Z)- | 1006.1 | 1004 | 17.3±9.98 | 59.25±33.98 | 4.43±3.2 | 4.84±0 |
|  | Glutaric acid, butyl isobutyl ester | 1659 | 1647 | 20.82±3.76 | 43.17±30.76 | 32.51±11.59 | 15.8±6.23 |
|  | Heptanoic acid, ethyl ester | 1097.9 | 1096 | 0.94±0.23 | 1.67±1.13 | n.d. | 0.83±0.3 |
| Fatty acid esters | n-Hexadecanoic acid | 1957.9 | 1978 | n.d. | 272.26±534.77 | 7.16±0 | n.d. |
|  | Octadecanoic acid | 2158.8 | 2177 | n.d. | 179.85±273.49 | n.d. | n.d. |
| Homoterpenes | DMNT | 1113.3 | 1107.5 | 7.05±4.76 | 7±7.77 | 4.93±1.52 | 3.8±3.79 |
| Ketones | 1-Octen-3-one* | 978.2 | 978 | n.d. | 7.46±3.37^a^ | 5.13±1.16^ab^ | n.d. |
|  | Cyclohexanone, 2,2,6-trimethyl- | 1035.4 | 1013 | n.d. | 3.23±1.13 | 3.57±2.47 | n.d. |
| Monoterpenes | α-Ionone* | 142.9 | 1425.6 | 0.4±0 | 14.52±9.71 | 6.27±6.18 | n.d. |
|  | α-Pinene | 925.5 | 936 | n.d. | 3.15±1.56 | 3.56±2.76 | n.d. |
|  | β-Cyclocitral* | 1219.1 | 1218.3 | 1.97±0^b^ | 6.1±3.92^a^ | 7.58±4.73^a^ | 1.97±0.87^b^ |
|  | β-Ionone* | 1478.2 | 1485.9 | n.d. | 7.6±5.92^a^ | 7.28±4.82^a^ | n.d. |
|  | δ-Carene | 1046.4 | 1011.3 | 2.94±2.07 | 19.89±32.2 | 21.54±29.14 | 0.65±0 |
|  | β-Homocyclocitral* | 1252.2 | 1236 | n.d. | 0.93±0.67^b^ | 1.38±0.78^a^ | n.d. |
|  | β-Pinene | 987.9 | 977.7 | n.d. | 13.27±0 | 6.64±4.55 | n.d. |
|  | Limonene | 1027.7 | 1029.5 | 1.26±0.85 | 15.33±10.05 | 15±21.01 | 3.12±2.13 |
|  | Eucalyptol* | 1028.6 | 1032 | 7.2±2.43^b^ | 30.01±21.47^ab^ | 100.88±114.12^a^ | 12.16±9.94^b^ |
|  | p-Cymene | 1023.7 | 1024 | 0.4±0 | 3.42±1.31 | 8.17±7.49 | 1±0 |
| Phenyl-propanoids | Cinnamaldehyde, (E)-* | 1272.3 | 1271.3 | n.d. | 21.81±15.65^a^ | n.d. | n.d. |
| Sesquiterpenes | α-Farnesene | 1495.6 | 1504.1 | n.d. | 115.84±189.68 | 22.05±14.22 | n.d. |
|  | δ-Cadinene | 1505 | 1533 | n.d. | 0.77±0.17 | 1.35±1.38 | n.d. |
|  | Unknown sesquiterpene* | 1477.5 | - | n.d. | 5.55±2.35^ab^ | 10.9±4.78^a^ | n.d. |
|  | γ-Gurjunene | 1589.6 | 1472 | 3.94±4.15 | 3.72±1.21 | 19.03±25.55 | 1.63±0 |
|  | α-Longipinene | 1485.9 | 1352 | 2.55±2.54 | 3.19±0.98 | 5.77±5.59 | 1.09±0.87 |
|  | δ-Elemene | 1332 | 1337 | n.d. | n.d. | 0.58±0.43 | n.d. |
|  | Germacrene-D* | 1481.3 | 1480 | n.d. | 6.23±2.76^a^ | n.d. | n.d. |
|  | Trans-caryophyllene* | 1416.2 | 1465 | 1.34±1.44^b^ | 7.68±6.86^a^ | 5.55±2.71^ab^ | 0.37±0.26^b^ |
| Terpenoids | Camphor | 1147.2 | 1143 | n.d. | 7.04±5.85 | 12.46±0 | n.d. |
|  | D-Carvone* | 1240.8 | 1242 | n.d. | n.d. | 1.17±0.92^a^ | n.d. |
|  | α-Terpineol* | 1192.1 | 1190 | n.d. | n.d. | 17.27±17.47^a^ | n.d. |
|  | Linalool | 1099.7 | 1084 | 1.54±0.08 | 37.79±0 | 4.07±3.88 | n.d. |
|  | p-Menthone | 1155.9 | 1150 | n.d. | 17.67±15.1 | 23.34±0 | n.d. |
|  | Terpinen-4-ol | 1178.2 | 1177 | n.d. | n.d. | 3.72±2.36 | n.d. |
